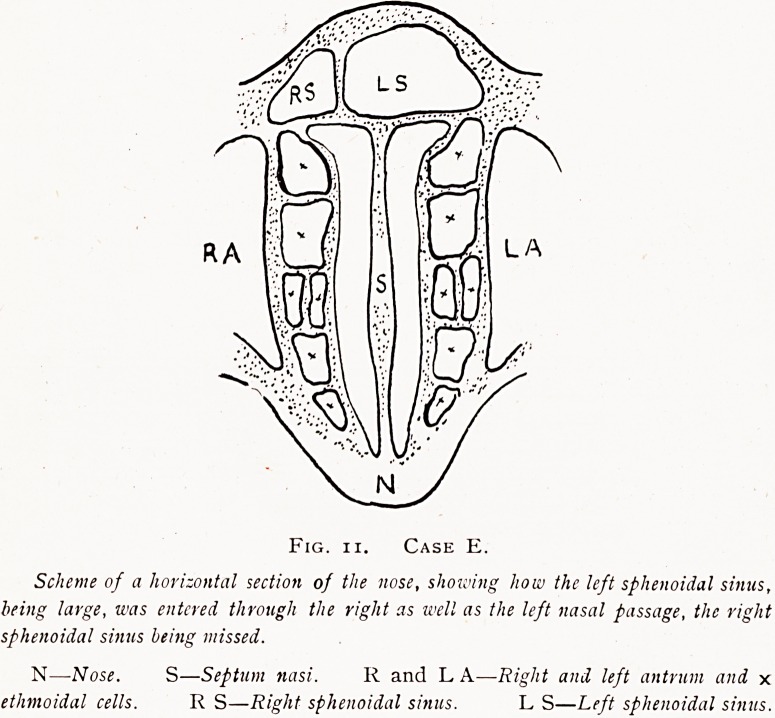# Notes on the Operation for Drainage of the Sphenoidal Sinus

**Published:** 1916-12

**Authors:** P. Watson-Williams

**Affiliations:** Major, R.A.M.C. (T.), Hon. Consultant for Diseases of the Ear, Nose and Throat to the Military Hospitals of the Southern Command; Lecturer on the Ear, Nose and Throat, University of Bristol, and in Charge of the Department for Diseases of the Ear, Nose and Throat, Bristol Royal Infirmary


### NOTES ON THE OPERATION FOR DRAINAGE OF THE SPHENOIDAL SINUS.

BY

P. WATSON-WILLIAMS,

*Major, R.A.M.C. (T.), Hon. Consultant for Diseases of the Ear, Nose and Throat to the Military Hospitals of the Southern Command;*

*Lecturer on the Ear, Nose and Throat, University of Bristol, and in charge of the Department for Diseases of the Ear, Nose and Throat, Bristol Royal Infirmary.*

---

In our previous issue the method of diagnosing the existence of infective sphenoidal sinusitis by means of the exploratory suction syringe was described, affording as it does the only criterion of the presence of the affection short of actual inspection of the discharge escaping from the sphenoidal sinus ostium, a very rare event compared with the relative frequency of the disease. Further, the possibility of clinching the diagnosis by a method which involves no removal of any normal structures is a great advantage over the more usual preliminary anterior middle turbinectomy; on this I laid stress in my foregoing communication. But, even when it is found necessary to expose the sphenoidal sinus by a free removal of the anterior wall, the normal middle turbinate body may be left untouched if one foregoes the doubtful advantage of operating under inspection, relying on the sense of touch and direction alone. Considering how imperfect inspection of the operative field must always be, however thoroughly and patiently the bleeding may be staunched or inhibited, after the partial removal of the middle turbinate, the sense of touch is in my experience a far more accurate and delicate guide.

By the method described below, I have opened several hundred sphenoidal cavities without any serious drawback, after practising it for over twenty-five years, to the exclusion of any other method, and although from time to time the instruments have been modified and I believe improved, my method of operating is substantially unaltered.

*Anæsthesia.*—While it is quite possible to operate under local anæsthesia, my preference is decidedly in favour of a general anæsthetic, ether followed by ether and chloroform. I always operate with the patient supine, lying flat on the back, but if preferred it is not difficult to operate in the sitting posture.

Preparation required consists in spraying or lavage of the nasal passages with some simple warm saline solution, followed by spraying with a solution of weak adrenal solution with cocaine, eucaine or novocain hydrochlorate, which serves the double purpose of shrinking the turbinals, reducing bleeding and the cardio-inhibitory reflex influence of the fifth nerve. Thus the preparation for operation is the same as for exploratory puncture.

The instruments I use are (1) an ordinary nasal speculum, (2) the blunt straight exploring cannula, (3) a small and a large size spheno-ethmoidal punch forceps. Both the cannula and forceps are marked in inches or half-inches from the tip backwards, so that one can always tell how far the ends are from the patient's nasal aperture or other landmark of the nose.

*Operation.*—If no exploration has been made beforehand for the diagnostic purposes it becomes the first step in the operation, firstly for establishing evidence of sphenoidal sinus disease, and secondly in order to measure the distance from some convenient landmark, such as the top of the patient's nose, to the anterior and to the posterior sphenoidal sinus wall, in a straight line passing between the nasal septum and the vertical plate of the middle turbinal as the cannula goes through the thin anterior sphenoidal sinus wall. Thus we may find that the anterior wall of the sinus is three inches from the nose tip and the posterior wall is four inches,

FIG. I.

*Showing Watson-Williams's sphenoidal sinus cannula passed through the thin anterior wall of the sinus so as to lie well within the sinus cavity, the "patient" lying on the back. The arrow shows the position at the normal sinus ostium, S.O.*

from which it follows that the sinus is at least one inch deep, less the thickness of the thin anterior wall. These distance measurements are invaluable data, as one knows exactly the distance of the anterior wall from, say, the nose tip and the depth of the sinus, so that one has only to pass the closed small forceps along the same line for a known distance, through the opening made by the cannula, and one knows for a certainty that the instrument is free inside the sinus. I then open the forceps and draw them forward till the opened blades are arrested by the posterior surface of the anterior wall, the male forceps blade being at right angles to the stem when opened, and therefore is larger, so to speak, than when closed. Then, closing the blades, one draws the instrument out about one-eighth of an inch or so, and on opening them again it

FIG. 2.

*Drawing to show the author's spheno-ethmoidal forceps cutting upwards and downwards in the removal of the anterior sphenoidal sinus wall.*

is found that the male blade is in front of the wall while the female blade is inside against the posterior wall, and thus when closure is made a piece of the wall is punched out. Keeping the forceps at the same distance in the sinus from the nose tip, the anterior sinus wall is quickly clipped away, upwards, outwards, inwards and downwards by rotating the forceps as one clips the wall away. In a straightforward case this can be done very rapidly, one minute sufficing to remove the whole face of the sinus. Although the description may suggest a tedious process, I may say that when timed I have made a right and left maxillary antrum exploration, an exploration of both sphenoidal sinuses, and the complete removal of the anterior wall in both sphenoidal sinuses, and at the same time opened the posterior ethmoidal cells, quite deliberately, and the whole procedure took under five minutes. Expedition was called for in this case, as the patient had a patch of quiescent broncho-pneumonia, due to bronchiectasis. Further information of value to the operator is obtainable by measuring the capacity of the sphenoidal sinuses. This may be done while the cannula is in the sinus in contact with the posterior wall in its deepest part as the patient lies supine, by throwing in normal saline solution with the syringe till the sinus is full, and then sucking the fluid contents into the graduated syringe.

It will be observed that owing to the oblique plane of the sphenoidal sinus anterior wall the obliquity of the cutting forceps when in position is equally suited for cutting upwards, and when reversed for cutting downwards, as well as in the inward and outward direction, provided the patient is on his back. If, however, the patient is in the sitting posture the special down-cutting forceps are more convenient for cutting downwards.

In cutting upwards one takes care to avoid pressure by the female blade against the roof of the sinus, although the rounded upper border of the female blade runs no risk of penetration without considerable force, and again in cutting outwards one should be cautious for fear of wounding the cavernous sinus, particularly when the sinus wall may be softened. But there is less fear in cutting freely inwards and downwards.

In the outward direction the sinus wall corresponds with the posterior wall of the posterior ethmoidal cells in most cases, and this portion should be removed by the larger size forceps, so as to get a very free opening of the sphenoidal sinus. Downwards the thicker part of the anterior wall may require the larger-sized and stronger forceps, but here care is required to obviate an injury to the internal branch of the nasal branch of the spheno-maxillary artery.

I always use the small-sized spheno-ethmoidal forceps in the first part of the procedure, and often complete the operation without using any large size. But after two or three clips with the small forceps, the orifice is large enough to take any of the larger sizes, and when one has finished the removal of the anterior wall, it is usual for the opening to be wide enough for any large-sized polypus forceps, *e.g.* Luc's largest size, to pass in in any diameter. One thus obtains very free opening of the sinus, without having to remove any portion of the middle turbinate. If the posterior ethmoidal cells have to be opened up, this is done by turning the back of the cutting forceps outwards and cutting just in front of the level of the anterior sphenoidal sinus wall, and working forwards as far as may be necessary, cutting open the inner cell walls in a similar fashion, but without removal of the anterior end of the middle turbinate. Of course, if there are special conditions rendering the removal or partial removal of the middle turbinal desirable, there is nothing to prevent one's doing so, but I never remove it *in order to* get access to the sphenoidal sinuses. Once a patient suffers from nasal accessory sinus disease requiring operative treatment, one never can foretell how much further removal or opening of sinuses may be eventually necessary ; it may be in years to come, and the preservation of the extensive surface of mucous membrane covering the inner and outer aspect of a middle turbinal may, if it be fairly healthy, prove of the greatest service in maintaining the essential physiological functions of the nose when perhaps the greater part of the ethmoidal labyrinth and all the other sinuses have had to be opened and drained. I do not wish to make a fetish of the middle turbinal, or to suggest that its partial removal is

FIG. 3.

*Specimen showing a polypus growing from the sphenoidal sinus and hanging down in contact with the posterior end of the middle turbinal body. The specimen gives a view of the outer wall of the left nasal passage, the nose to the right, the body of the sphenoid to the left.*

always to be avoided, or that such removal must necessarily lead to discomfort. On the contrary, I, in common with every rhinological surgeon, unhesitatingly remove considerable portions of the turbinal when it is diseased, and in some cases even when, although healthy, yet owing to its anatomical form or relationships, is a source of trouble. But to remove such an important physiological structure as the middle turbinal as a matter of routine and merely to facilitate operation on other structures and sinuses, and when one can avoid doing so, is, I hold, unsound practice; and the desirability of more conservative methods is not rarely emphasised by the irreparable damage to a patient's nasal passages, leading to crusting, dryness of the pharyngeal mucosa and so forth, examples of which come to the notice of every practising rhinologist.

Polypoid degeneration of the sinus mucosa may call for curettage, and I usually prefer to reserve this for after-treatment under cocaine anæsthesia. One may use a very small, sharp ring curette for the purpose, and though it may be firmly used against the floor and inner wall, the greatest caution should of course be observed if it is applied to the outer wall or roof of the sinus. When, as rarely happens, polypus has formed in the sinus, it is better to pick them out with blunt polypus forceps.

Special operative procedures may be considered desirable in the following circumstances:—

(*a*) Where there is a difficulty in preventing the opening into the sinus from contracting. Where the sinus is of an average capacity, and the outer portion of the anterior wall corresponding to the posterior ethmoidal cell has been removed, as well as the free inner portion facing the nasal passage, such closure is rarely a cause of trouble. But in patients whose sphenoidal sinuses are small, and one side only is affected, one may obtain a constant free opening by removing the anterior part of the sphenoidal sinus floor, either with a chisel or with strong cutting punch-forceps. When both sinuses have to be opened, I think a better procedure is to remove the posterior half-inch of the nasal septum above the choanæ narium, and then continue the removal backwards, so as to remove most of the inter-sphenoidal sinus septum, and thus throw both cavities into one. Or again, one may sometimes gain one's end by breaking down the septum between the two sinuses by means of a bent, small, round-ended rasp, and then nibbling away the rest of the septum.

FIG. 4.

*Illustrating the author's method of removing the posterior part of the nasal septum, as well as the inter-sphenoidal sinus septum, in special cases.*

(*b*) When both sinuses are diseased and their development is irregular. Thus it is not rarely found that the sphenoidal sinus of one side is much larger than the other, so that the larger sinus extends well over to the opposite side, being over-developed at the expense of the other smaller sinus. In such cases, *e.g.* with a large left sphenoidal sinus, the left sinus may have been opened on both sides, and the smaller right sinus overlooked, inasmuch as the second opening made into the large left sinus through the right nasal paassge may be readily mistaken for an entrance to the small right sphenoidal sinus, pushed, so to speak, well to the outer side of the right nasal passage. But of course if this small right sinus is infected and suppurating it must be opened, and under such conditions it is better to clear away the intersinus septum and practically make one large common sphenoidal

FIG. 5.

*Transverse section through the sphenoidal sinuses. The dividing septum is displaced to the left, the right sinus being large, and it would be possible to enter this sphenoidal sinus through either nasal passage.* (*Sieur et Jacob.*)

sinus, otherwise the smaller sinus is difficult to keep well opened.

The posterior ethmoidal cell is similarly a source of difficulty, though even more rarely, when it develops above the sphenoidal sinus proper, that is, when it clinically forms a false sphenoidal sinus above the true one. The floor of the irregularly-developed ethmoidal cell then forms a roof to the true one, and this thin intervening more or less horizontal septum may have to be clipped away.

*Dangers to avoid in operating on a sphenoidal sinus.*—One need scarcely allude to the disasters that ensue from missing the sphenoidal sinus and taking a direction upwards to the roof of the nasal passage in front of the sinus, since if no force be used and only a blunt exploring cannula is employed, and the cannula is made to impinge on the anterior surface of the sinus according to the directions given in my preceding note, one cannot do any harm. If the sphenoidal sinus is small or non-existent, there is all the greater thickness of the body of the sphenoid between the nose and the foramen magnum. But when the sinus is

FIG. 6.

*Section showing an unusually large and well-developed sphenoidal sinus which occupies almost the entire sphenoidal body and extends backwards so as to leave but a very thin plate of bone between the sinus and the foramen magnum. CO, optic canal; CI, prominence formed by the carotid canal on the outer wall of the sinus.* (*Sieur et Jacob.*)

exceptionally large and deep, the cavity may extend backwards so that only a very thin plate of bone separates the sinus from the medulla oblongata and, very rarely it is true, there may be nothing but periosteum and mucosa, with no bone at all. For this reason when one has passed the anterior sphenoidal sinus wall and the cannula goes back four inches in an adult, one must naturally avoid using any force, and carefully feel for the impinging of the distal end against the posterior wall. But in removing the anterior wall one need never go so far back, and the larger the sinus the less the need for touching the posterior wall.

FIG. 7.

*Section of the left side of the head showing a well-developed sphenoidal sinus through which the left optic canal forms a complete tube containing the optic nerve, which is very liable to serious injury by instruments passed into the sinus (Onodi).*

The canal for the optic nerve very frequently runs along the upper and outer walls of the sphenoidal sinus, and may form a very thin bony partition, or the canal with the contained optic nerve may even form a thin tube traversing the outer and upper part of the sphenoidal sinus. The danger of injury to the nerve and consequent loss of sight from rough usage of blunt instruments in the sinus cavity, and the grave risks involved in the introduction of any sharp instrument, need hardly be further emphasised. And when the sinus has long been involved in a chronic suppuration, the possibility of softening of these thin bone partitions must always be remembered.

The outer wall, corresponding with and forming the inner boundary of the cavernous sinus, must be treated with great caution and respect. I have never seen any case reported where the cavernous sinus has been wounded, but I believe this has occurred, and naturally with fatal result.

Considerable trouble may arise from primary or secondary

FIG. 8.

*Section of the left side of the head, showing a large sphenoidal sinus with a transverse prominence, the optic canal, and more posteriorly a vertical prominence, the canal for the carotid artery.*

hemorrhage from the sphenopalatine artery, as it crosses beneath the lower margin of the anterior sphenoidal wall to reach the nasal septum, where it becomes the posterior septal artery. Injury to the artery is generally the result of removing the thick lower margin of the anterior wall, and is more likely to cause trouble if the vessel is only partly divided. Hemorrhage from this source can almost always be arrested by drawing a well-fitting postnasal plug of gauze very firmly into the posterior choana, and packing with a strip of half-inch gauze firmly against the remains of the sinus wall. In one patient under my care it became necessary to ligate the external carotid artery when the hemorrhage at once ceased; but before this was done I had tried plugging and packing the nasal passage more than once, with the result that each time the plug was removed the hemorrhage recurred. This patient had very high arterial tension. Hemorrhage from this source must always be reckoned on as a possibility, and may occur for the first time several days or even a week after the operation. I did not meet with any case of troublesome hemorrhage till I had operated on over two hundred sinuses, and after coming to the conclusion that I had rather over-estimated it as a possible occurrence, but that view I no longer hold. In the first six months of the current year I find that in my private practice I operated on twenty-eight sphenoidal sinuses in eighteen patients, and in one hemorrhage occurred, requiring plugging and packing a few hours after the operation. Of the patients operated on in my hospital clinic no case required packing, and I should estimate that not more than one per cent. is attended with troublesome primary or secondary hemorrhage.

*After treatment* consists in lavage of the opened sinus at first on alternate days and subsequently daily, till the purulent secretions diminish or disappear. In some cases, and where the discharge persists for more than a fortnight, it is well to mop out the sinus cavity with some strong antiseptic, such as colloidal silver solution or iodine. Especially for the first week after operation it is well to spray the nose with cocaine solution before lavage, as it lessens the swelling of the middle turbinal mucosa, and while making the passage less uncomfortable for the patient, this also facilitates the introduction of the cannula and the escape of secretion. For lavage of the sinus I use a No. 1 or No. 2 short silver Eustachian Catheter, which fits on to the nozzle of a rubber syringe.

As a few illustrative examples of cases recently operated on for sphenoidal sinus disease, the following notes may prove useful. A, B and C are examples of *latent sinus infection,—Symptoms Marked, Signs Indefinite.*

A.—Miss T. H., aged 26, referred by Mr. Last, complained of some postnasal discharge and irritation at the back of the nose for several years, and for two years of giddiness also, and more recently a constant feeling of lassitude. She was thin and apathetic, had noticed her memory for daily events had grown very unreliable, with decreasing power of mental concentration; at times great mental depression. Found her eyesight weak if she used it much; had seen Mr. F. R. Cross, who found refractive errors, for which glasses were ordered. Latterly, too, digestion weak. Was referred by her medical attendant to a physician, who found her neurasthenic. Two and a half years previously had an attack of influenza, and the symptoms were said to have been noticed mainly for two years.

Examination of the nasal passages by ordinary methods revealed nothing abnormal, except some doubtful increased redness of the mucosa. Transillumination and exploration of the right maxillary antrum negative. A nasal spray with a simple saline solution ordered night and morning. She improved very much.

Nine months later she was again referred to me with all the old symptoms. Under an anæsthetic her maxillary antra and sphenoidal sinuses were explored by the suction syringe. Both antra were quite clear, but the sphenoidal sinus contained a little muco-purulent secretion. The latter on culture yielded a good growth of G.P. streptococcus brevis, also G.P. streptobacillus and G.P. staphylococcus.

Measurements:—Right: nose-tip to anterior wall of sinus 3 inches; to posterior wall, $3\frac{5}{8}$ inches. Left: nose-tip to anterior wall, 3 inches; to posterior wall, 4 inches.

They were large sinuses, from $\frac{5}{8}$ to 1 inch at least in depth, and the anterior wall was freely removed on both sides, and the posterior ethmoidal cells were likewise opened.

The patient left the home on the eighth day after the operation, with the "head feeling clearer and much brighter mentally." She continued to improve steadily, and a month later resumed her occupation as an author.

In this case the sphenoidal sinuses were apparently anatomically normal.

B.—Mr. J. W. S., aged 30, referred to me by Lieut.-Colonel Munro Smith, complaining of a feeling of a lump in the throat and a constant soreness, and some left frontal headache. He was mentally duller than his normal standard, and found his organising work very difficult to carry through. Some years previously he underwent an operation by Sir Felix Semon for nasal obstruction, and subsequently was again operated on by another rhinologist, as the sense of obstruction was not wholly removed. When I saw him the nasal passages were, if anything, too freely patent. But endo-rhinoscopic examination showed that the middle turbinals were boggy and almost polypoid in aspect, and strings of muco-pus were seen above his left Eustachian tube. He had also granular pharyngitis.

By the suction syringe the left maxillary antrum was found to contain no abnormal secretions; I advised against any operation, and ordered a nasal spray. Six months later he felt no better, although he had felt relief at first from the spray; and now complained not only that he felt as tired as ever, but that he felt giddy, his memory bad, with difficulty in mental concentration, and post-nasal discharge in the morning. Operative treatment then advised.

Further exploration of the maxillary antra showed that they were healthy. Both sphenoidal sinuses measured 4 inches from the nose-tip to the posterior wall. They were freely opened, as well as the posterior ethmoidal cells, and also the left frontal sinus, by my usual pernasal method. He made a quick recovery, and after a short course of lavage of all the opened sinuses, became free from all symptoms and recovered his mental alertness.

The sphenoidal sinuses were peculiarly shallow in vertical measurement, but running backwards a full inch from the anterior wall, and the anterior walls were unusually thick.

C.—A similar kind of case as regards symptoms and results. But in this patient one encountered difficulties from the fact that the posterior ethmoidal cells encroached on the sphenoidal sinus body, and were at first mistaken for the sphenoidal sinuses lying more inferiorly (as shown in diagram). These posterior ethmoidal cells were opened with the sphenoidal sinuses and all thrown into one cavity.

FIG. 9. CASE C.

*Ethmoidal cell lying above the sphenoidal sinus, and at first leading to its being mistaken for the sphenoidal sinus.*

### *Sphenoidal sinus Suppuration copious, Symptoms Absent.*

D.—Captain L., aged 40, was on active service in Egypt, and had been suffering from morning headaches and purulent discharge from the left nasal passage anteriorly and postnasally, with occasional cacosmia (subjective bad smell). Two years previously he had a left radical antral operation at Bendigo, followed by vaccines for his frontal sinus infection, and for a time was much relieved. He was very hoarse, almost aphonic, when I saw him, and as he formerly had tuberculous lungs, was afraid that he was developing laryngeal tuberculosis. His complexion was ruddy, but he looked thin and suggestive of a tuberculous infection. Examination of the lungs and sputum did not support the suggestion of tuberculous recrudescence.

A skiagram by Mr. Bergin showed that the left frontal sinus was diseased, the right normal. Both sinuses were very large; the left antrum was dull. The sphenoidal sinuses were well brought out in the skiagram, but though large, appeared quite clear.

I advised a pernasal frontal sinus operation, with a preliminary exploration of the other sinuses whilst under the anæsthetic, not that I expected to find anything definite. It is only fair to state that I had not carefully investigated the patient for sphenoidal sinus trouble, inasmuch as operation from the left frontal sinus was undoubtedly necessary, and the method of investigating the sphenoidal sinuses whilst the patient is under the anæsthetic suffices to clear up any doubts one might have; and moreover he had *no symptoms suggesting sphenoidal sinus infection* to me, the sequel is therefore highly instructive.

On exploration by the suction syringe, one found :—
Right antrum, clear.
Left antrum (formerly operated on), pus.
Right sphenoidal sinus, copious thick pus.
Left sphenoidal sinus, copious thick pus.

So copious was the pus it simply oozed out of the proximal end of the sphenoidal sinus cannula on each side as soon as the style was removed, and more was drawn into the syringe for culture. The posterior sphenoidal sinus walls felt boggy and unhealthy.

Measurements showed that the right sinus posterior wall was $4\frac{1}{16}$ inches from the nose tip, and on the left 4 inches. The two sphenoidal sinuses communicated with each other, probably the inter-sinus septum had become partly absorbed.

The operation was completed by removing the anterior sphenoidal sinus walls, opening the posterior ethmoidal cells on both sides, making the opening of the left maxillary antrum into the nose more free, especially towards the front, and doing a pernasal frontal sinus operation so that a bougie 19 mm. in circumference dropped readily and freely into the frontal sinus.

A report by Professor Walker Hall gave the following results :—

Right antrum. Film examination : No polynuclears, no cocci. Culture : Feeble growth of G.P. staphylococcus.

Sphenoidal sinuses. Film : Masses of polynuclears and numerous streptococci. Slight phagocytosis. Culture : G.P. streptococcus longus.

The patient rapidly recovered, and with daily lavage in a short period was very much better and stronger. Two months later he still had some post-nasal discharge, but had lost all headache and gained weight. He had no difficulty in washing out his own frontal and sphenoidal sinuses.

*Symptoms Marked, Signs Definite (blindness, etc.).*

E.—Mr. D. H. M., aged 28, referred by Mr. Cross, as the patient had complete blindness in his left eye without any detected cause, unless it was nasal.

The patient had neuralgic headaches following slight influenza a month before, and a month later complained of blurred vision in the left eye for one day, and then left eye blindness. He said the pains in the head had been very severe. The patient assured me that he had no discharge from his nose, and that there had been none before I saw him.

Examination of the nasal passages by the usual methods revealed no discharge or any diseased condition, but by the endo-rhinoscope a little muco-pus was seen in the roof of the nasal passages on both sides. See Fig. 10. Operation advised.

FIG. 10. CASE E.

*Endo-rhinoscopic condition. In median line one sees the posterior border of the nasal septum, and on either side the posterior ends of the middle turbinals, and below them, just coming into view, the posterior ends of the inferior turbinals. A streak of muco-pus stretches from the septum upwards to the posterior wall of the pharynx.*

On exploring the sphenoidal sinuses (under local anæsthesia) one obtained the following results :—

Right sphenoidal sinus, $3\frac{1}{8}$ inch, nose-tip to posterior wall.

Left sphenoidal sinus, $3\frac{1}{2}$ inch, nose-tip to posterior wall.

The posterior walls of the sinuses felt velvety, and on the right side polypoid. From the left sinus a definite blob of white pus was extracted by the syringe, and from the right thick, bloody muco-pus.

Both sphenoidal sinuses were opened, and the posterior ethmoidal cells also. An opening into the frontal sinuses sufficient for lavage was easily made by removing the fronto-ethmoidal cells.

From the right sphenoidal sinus strepto-diplococci were grown in culture ; the left sinus yielded no growth.

One of many interesting points in the last-mentioned case to which attention is invited is that further investigation showed that this patient had a very large left sphenoidal sinus, and one had entered this left sphenoidal sinus first through the right nasal passage, and subsequently again through its own left side, and *the right sphenoidal sinus had not been entered at all.* Consequently the culture attributed to the right sinus came from the left, and apparently the washing out of the sinus had rendered it so relatively clear that the left side specimen appeared sterile. This explained the fact that the left eye was blind when apparently only the right sinus was involved. Of course, the fact that the left eye seemed rendered blind by a right sphenoidal sinus was

FIG. 11. CASE E.

*Scheme of a horizontal section of the nose, showing how the left sphenoidal sinus, being large, was entered through the right as well as the left nasal passage, the right sphenoidal sinus being missed.*

N—*Nose.* S—*Septum nasi.* R and L A—*Right and left antrum and* x *ethmoidal cells.* R S—*Right sphenoidal sinus.* L S—*Left sphenoidal sinus.*

capable of explanation by other means, but the case is cited as an example of sources of error due to anatomical irregularity, and to show the necessity of assuring oneself as to which sphenoidal sinus is entered, and not assuming that because one has entered a sphenoidal sinus through one side of the nose that the cavity properly belongs to that side.

The case also presents many points of great interest in its ocular aspects, but these I do not propose to enter into here, reserving such details for a further contribution on the sphenoidal sinus and ocular defects. Suffice it to say that unfortunately the blindness became permanent, as the operation was not in time to save the sight, though in other patients operation on the sphenoidal sinuses restored vision. But the general condition of the patient was very much improved. The case illustrates the importance of investigating the condition of the sinuses when defective vision is not explicable by conditions other than sinus infection.

Taking these four examples of sphenoidal sinus infection together, it is a striking fact that the one patient in whom the sinuses were full to overflowing with pus had no symptoms (apart from signs) suggesting that they were the seat of disease, while the other three patients, who all had marked symptoms, had very little pus in the sphenoidal sinuses, which nevertheless were infected and diseased. I suggest that in the numerous polynuclears in the one case, as contrasted with paucity of polynuclears in the others, we have an explanation of the remarkable differentiation to which I have drawn attention.

## Figures and Tables

**Fig. 1. f1:**
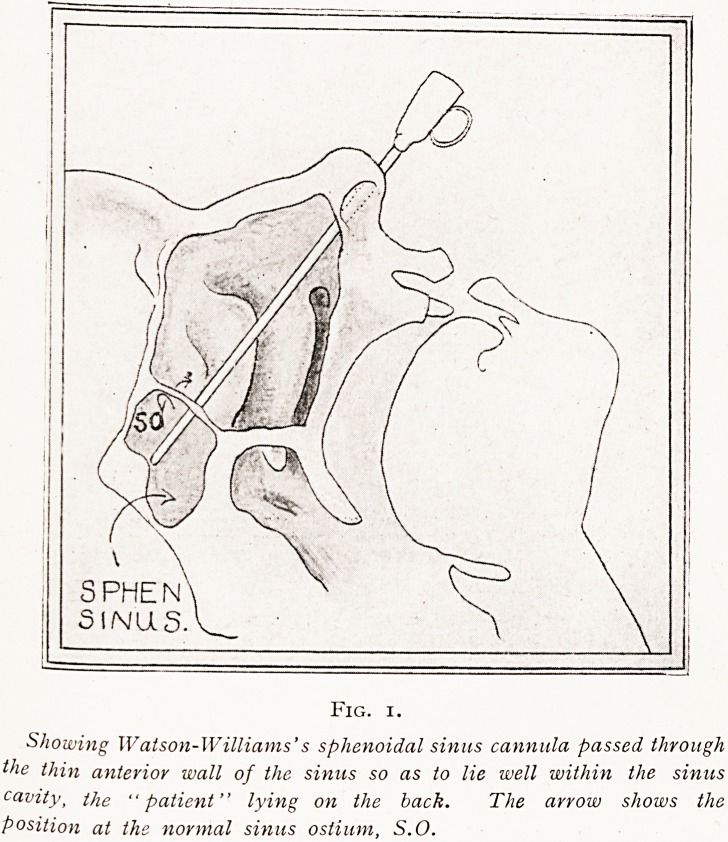


**Fig. 2. f2:**
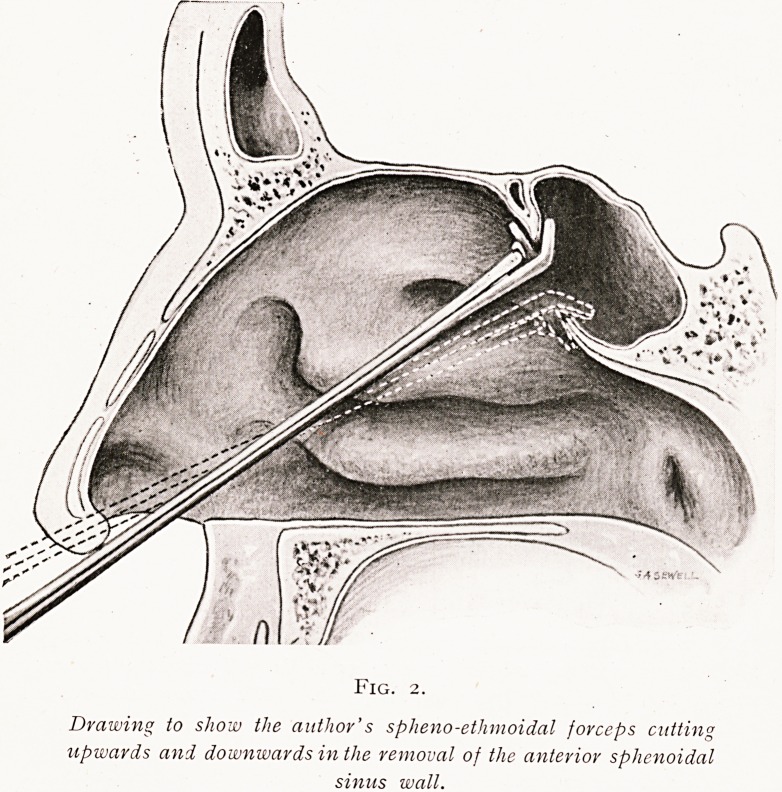


**Fig. 3. f3:**
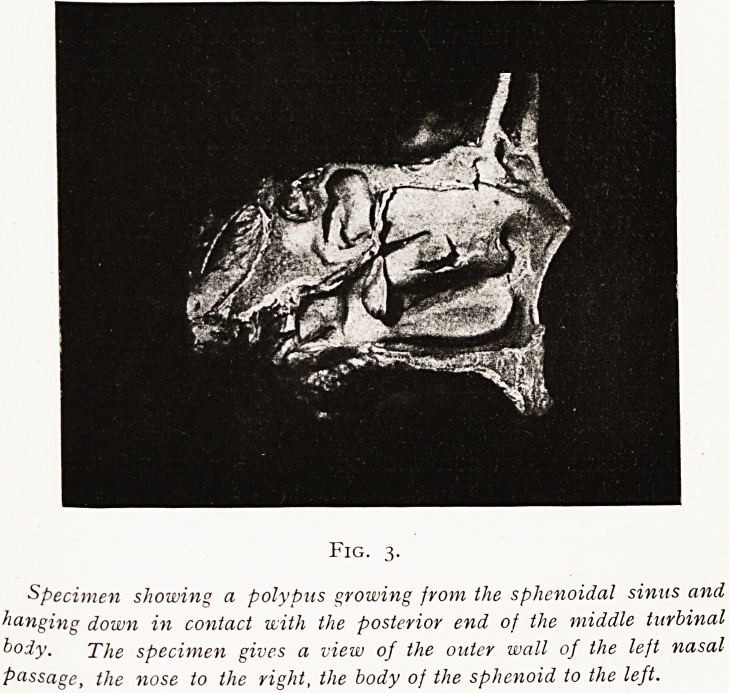


**Fig. 4. f4:**
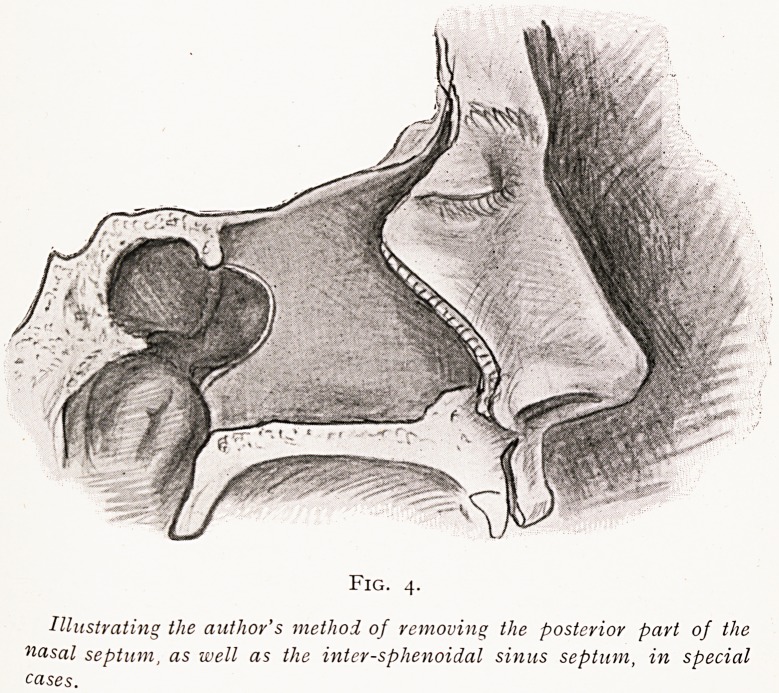


**Fig. 5. f5:**
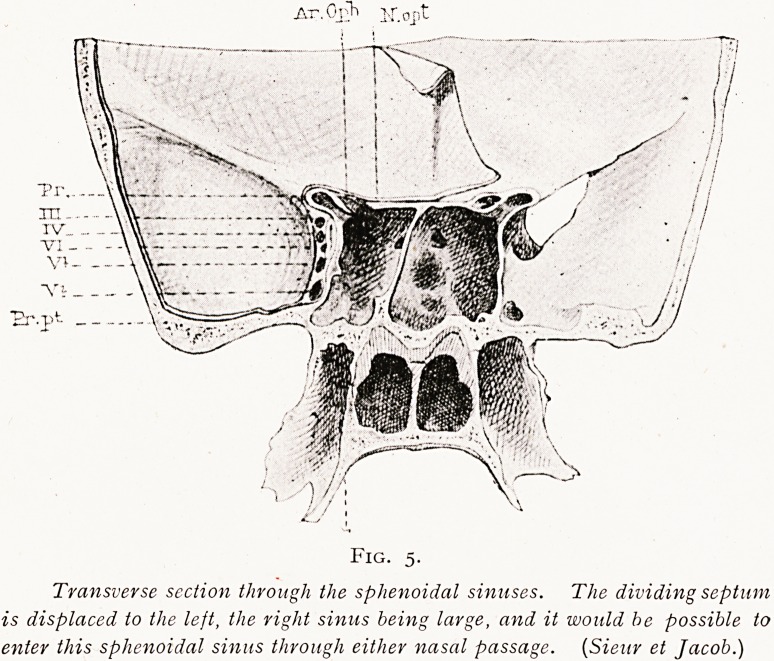


**Fig. 6. f6:**
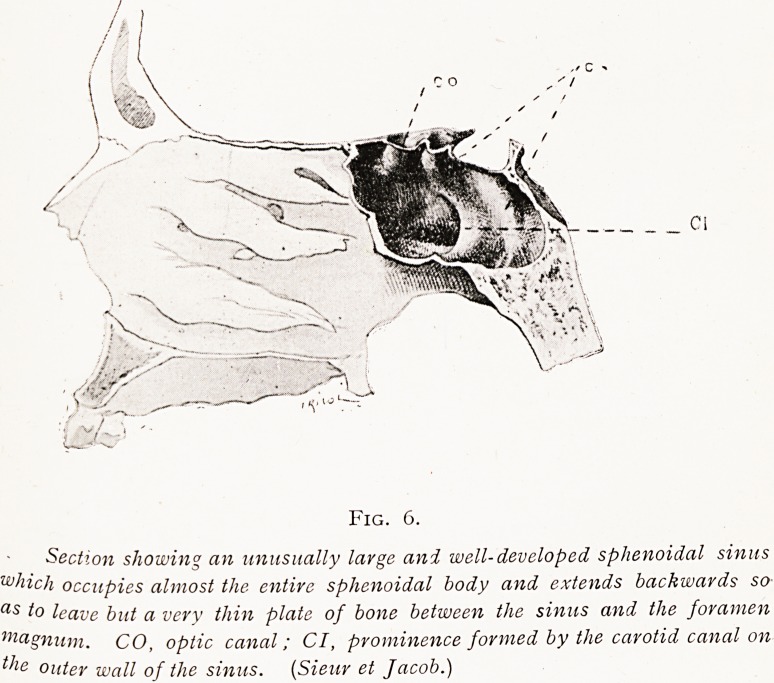


**Fig. 7. f7:**
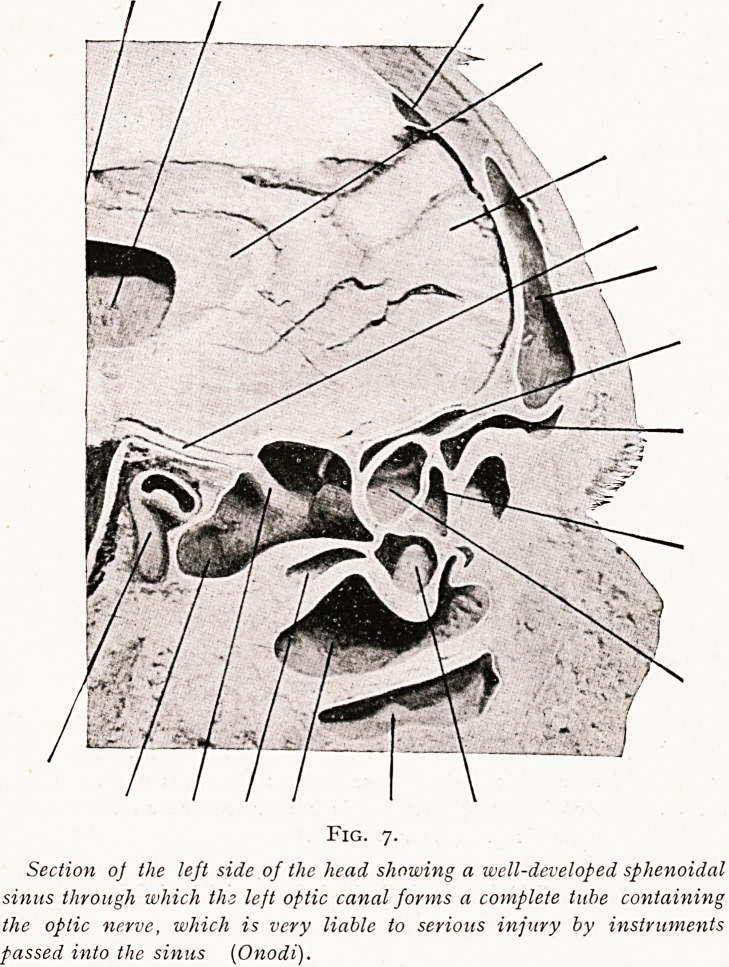


**Fig. 8. f8:**
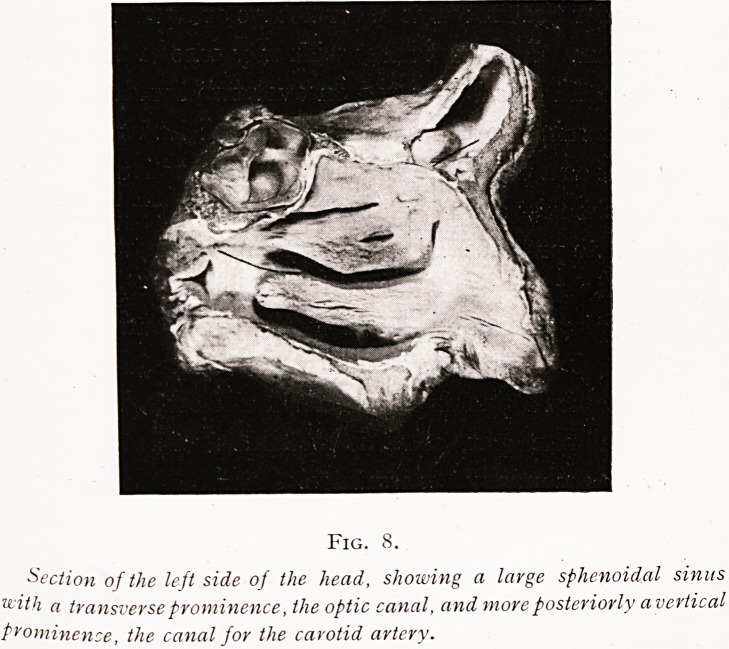


**Fig. 9. Case C. f9:**
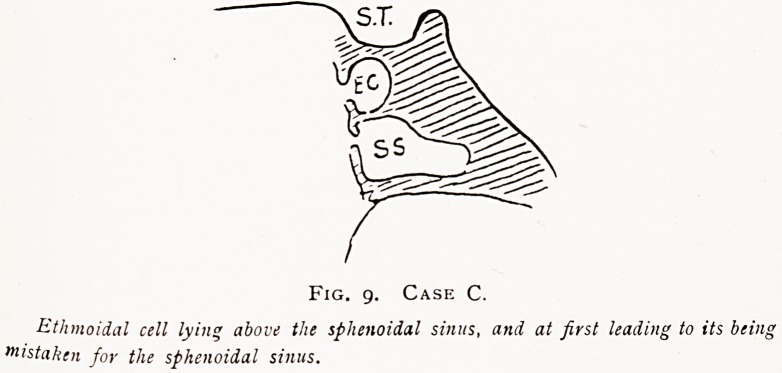


**Fig. 10. Case E. f10:**
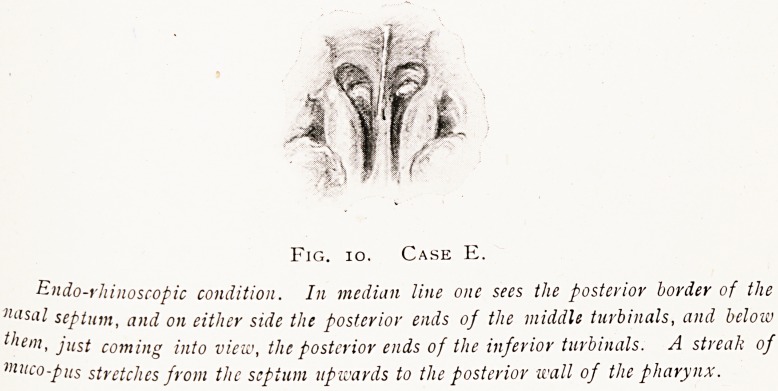


**Fig. 11. Case E. f11:**